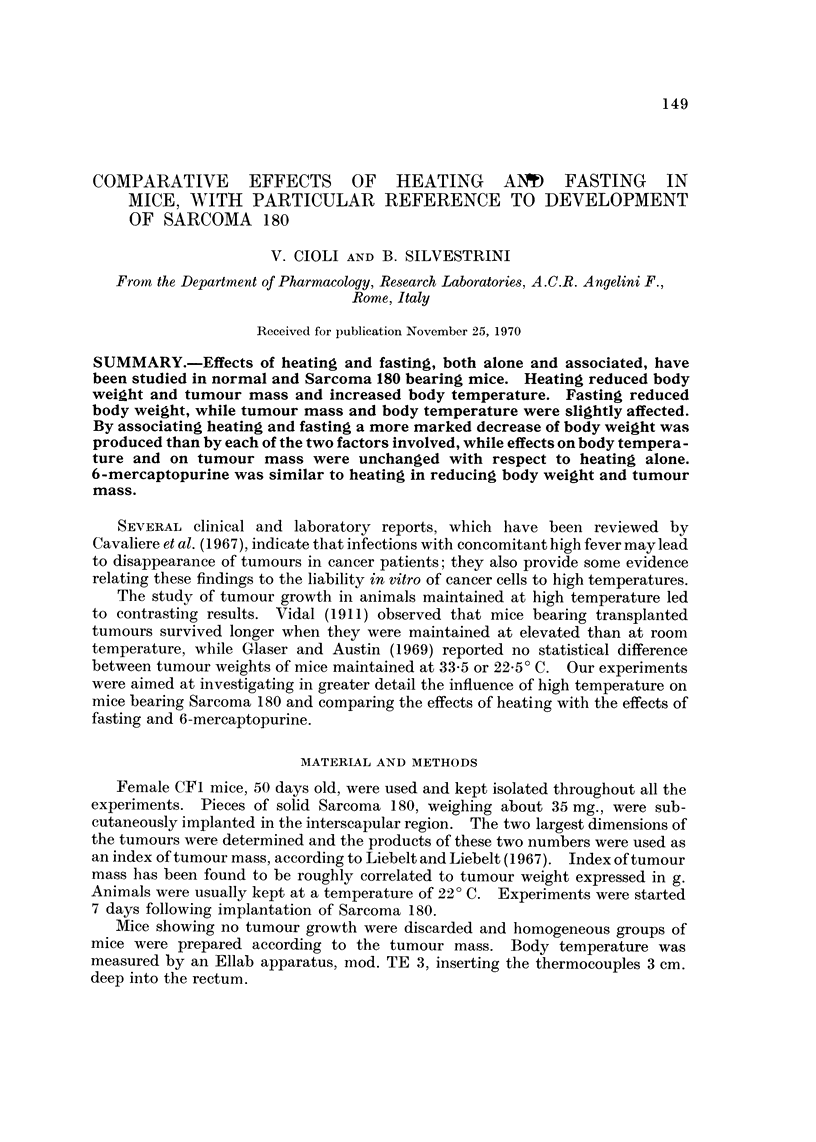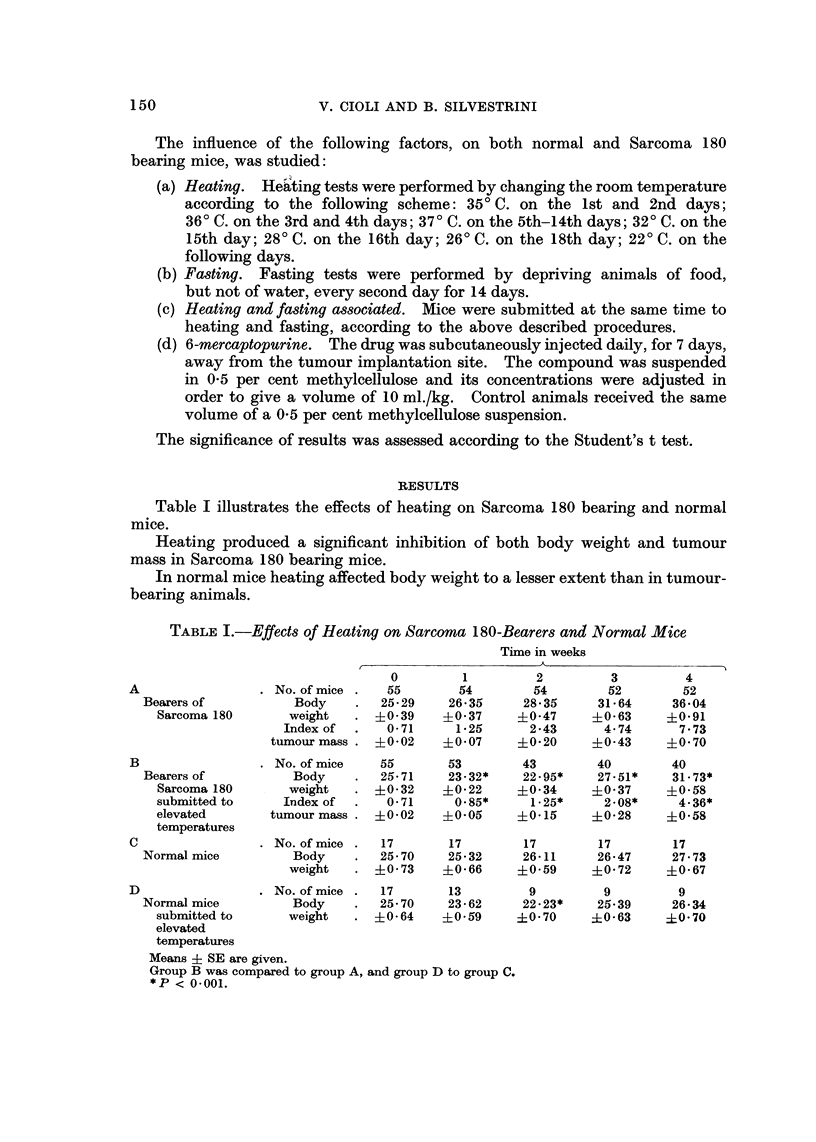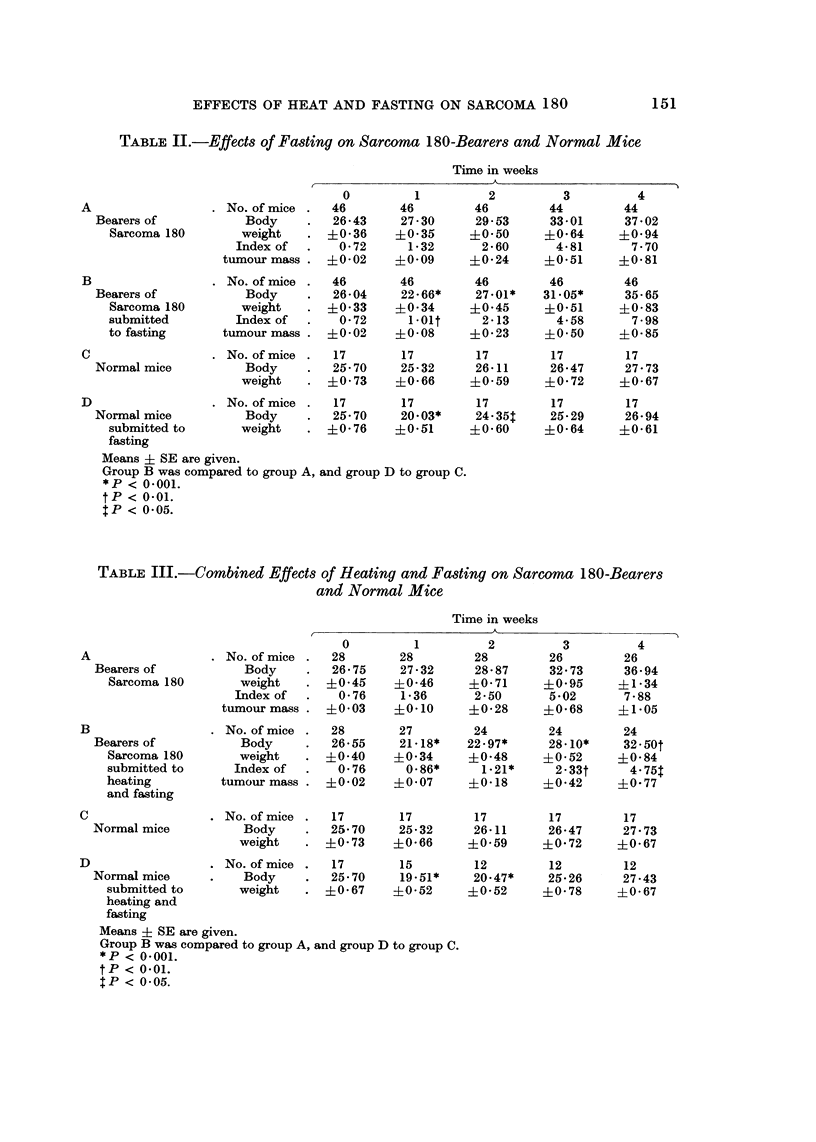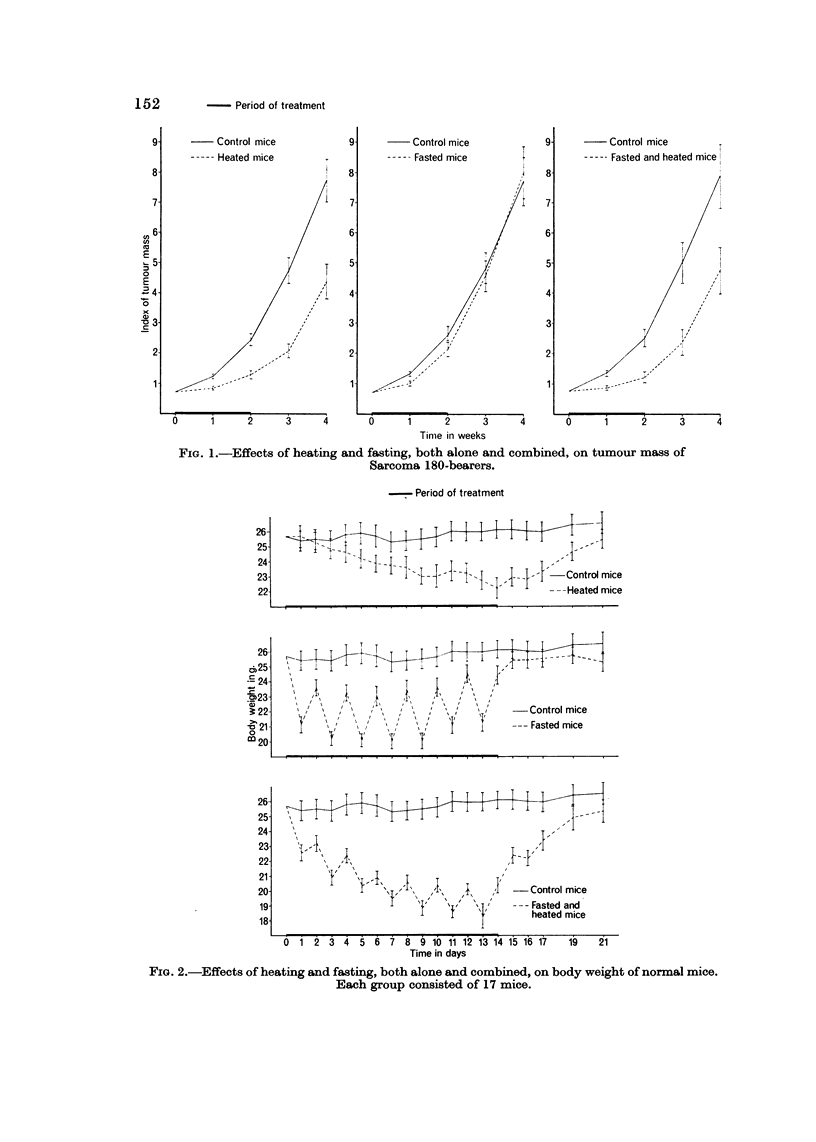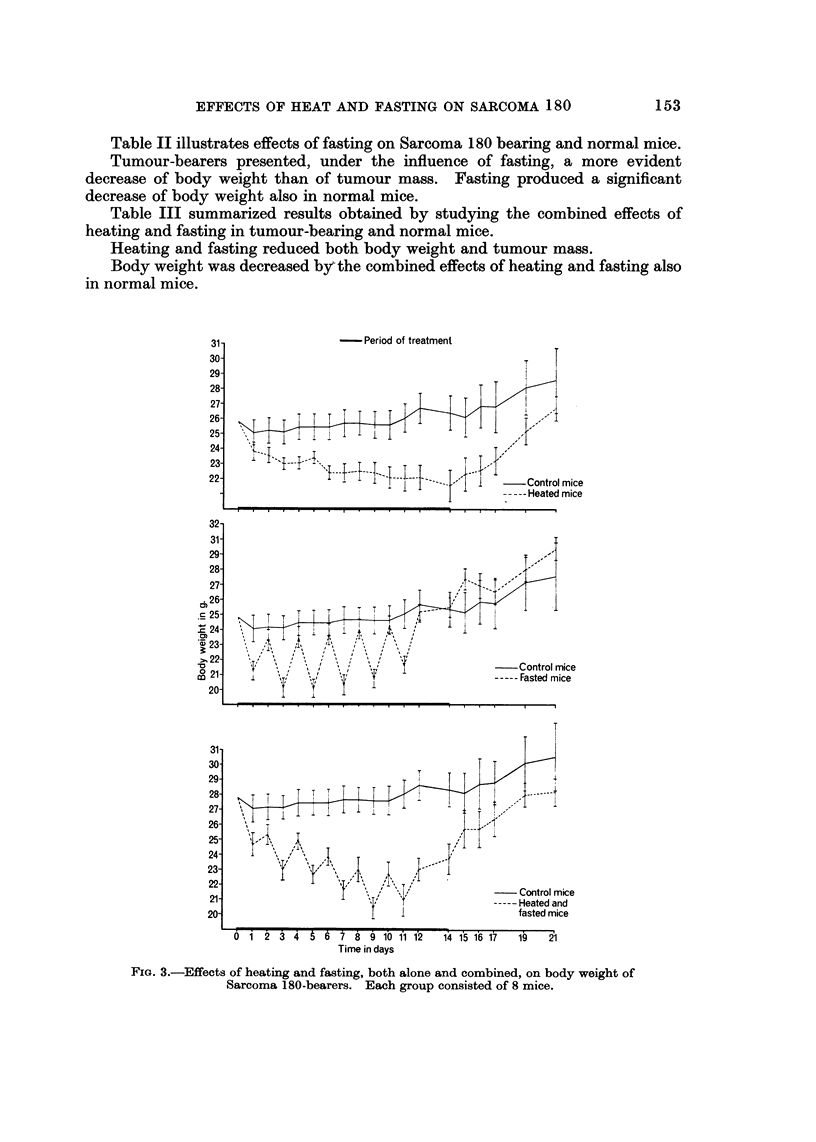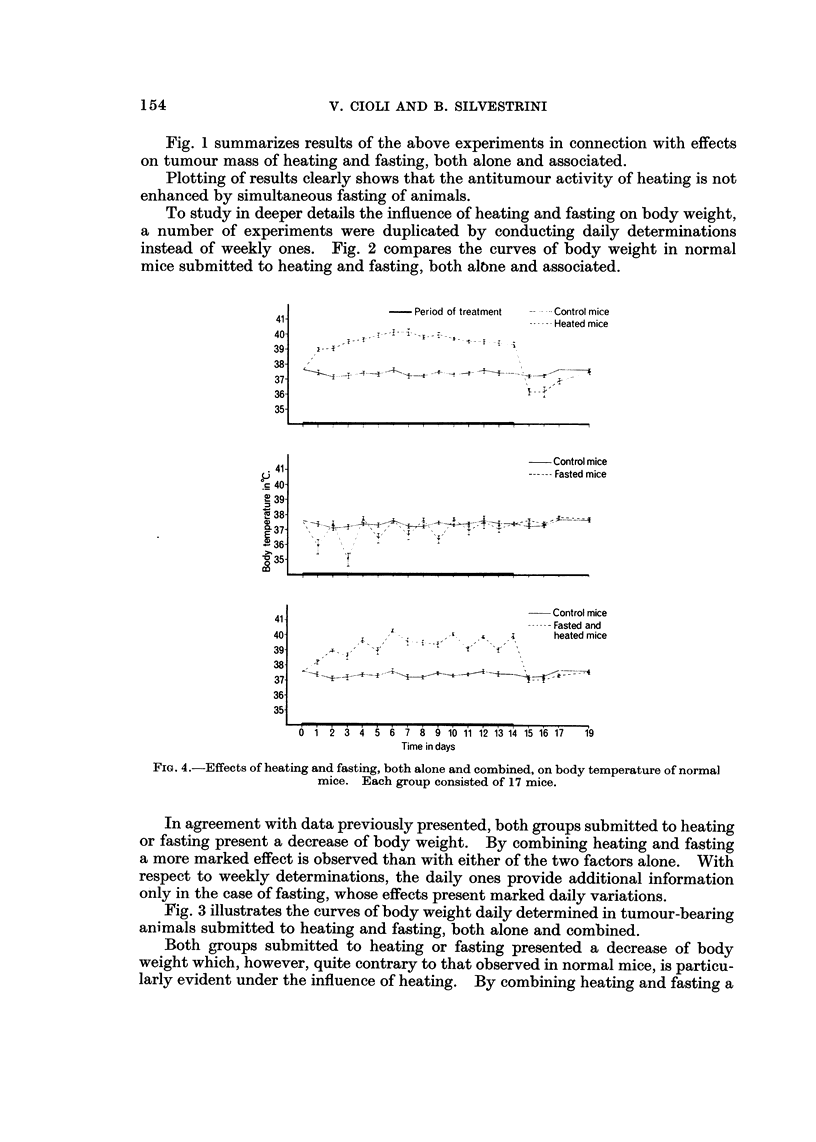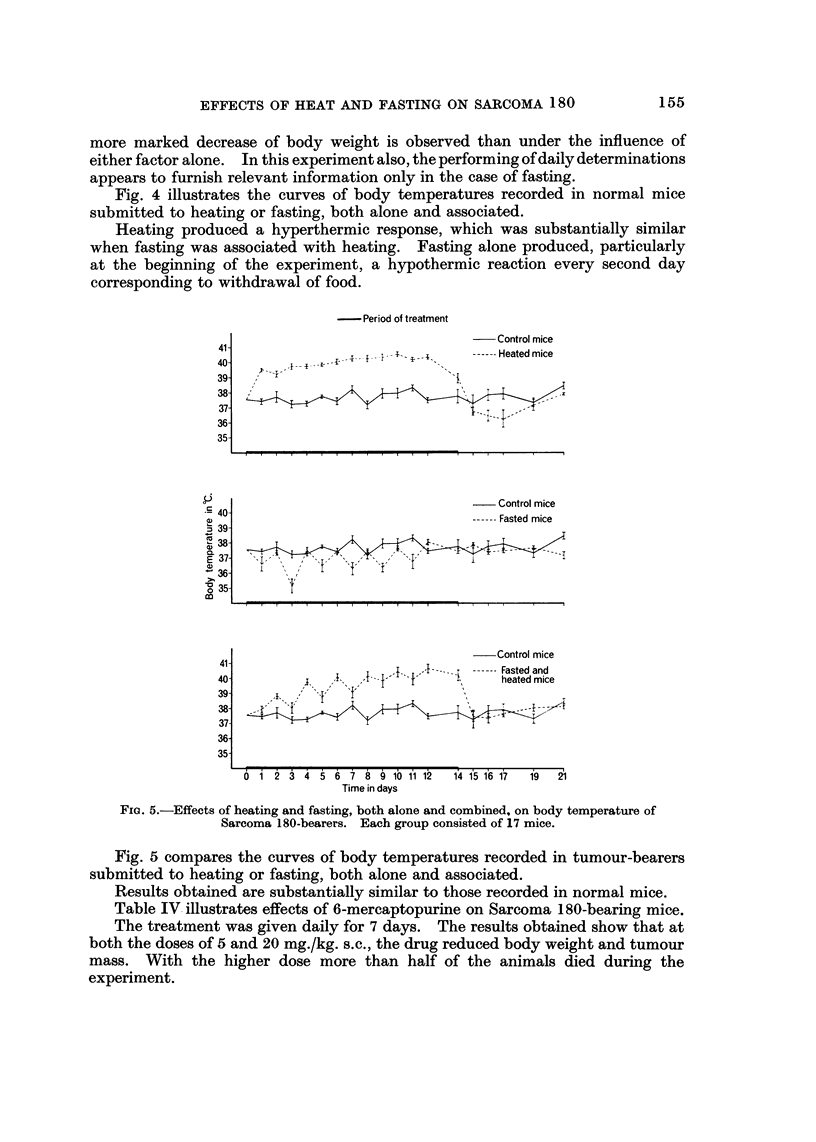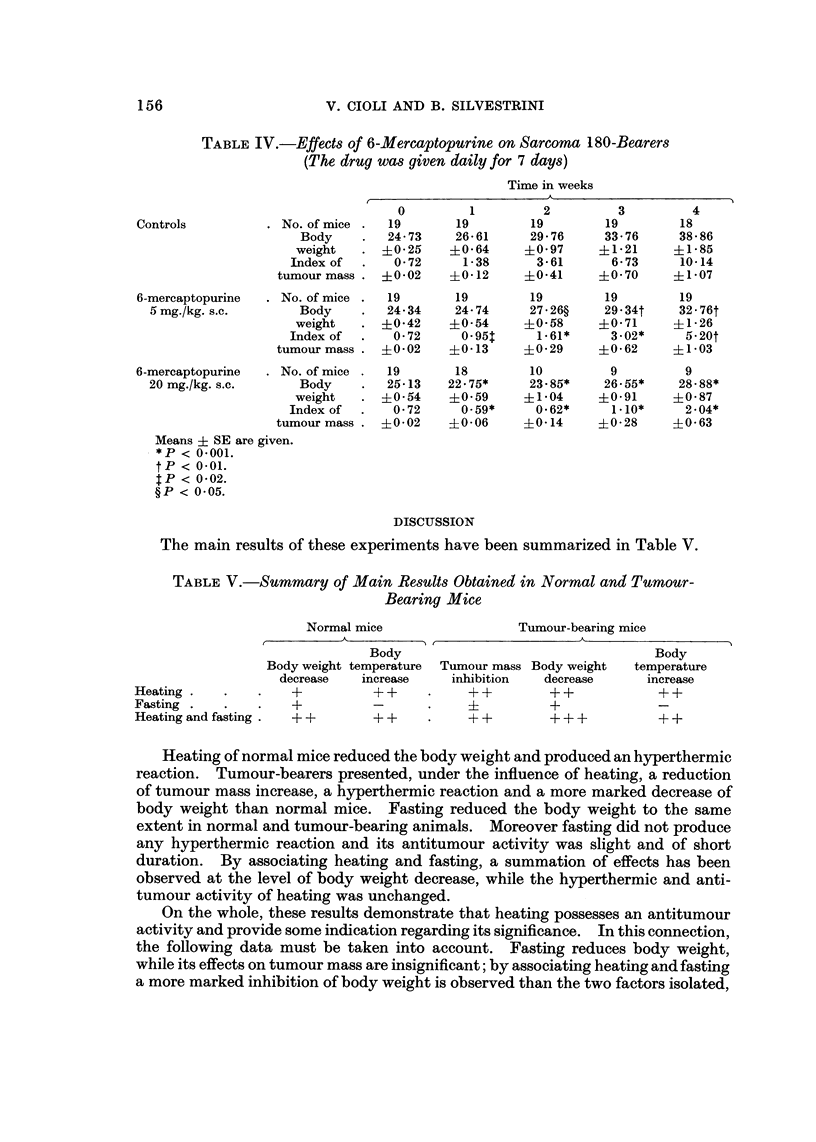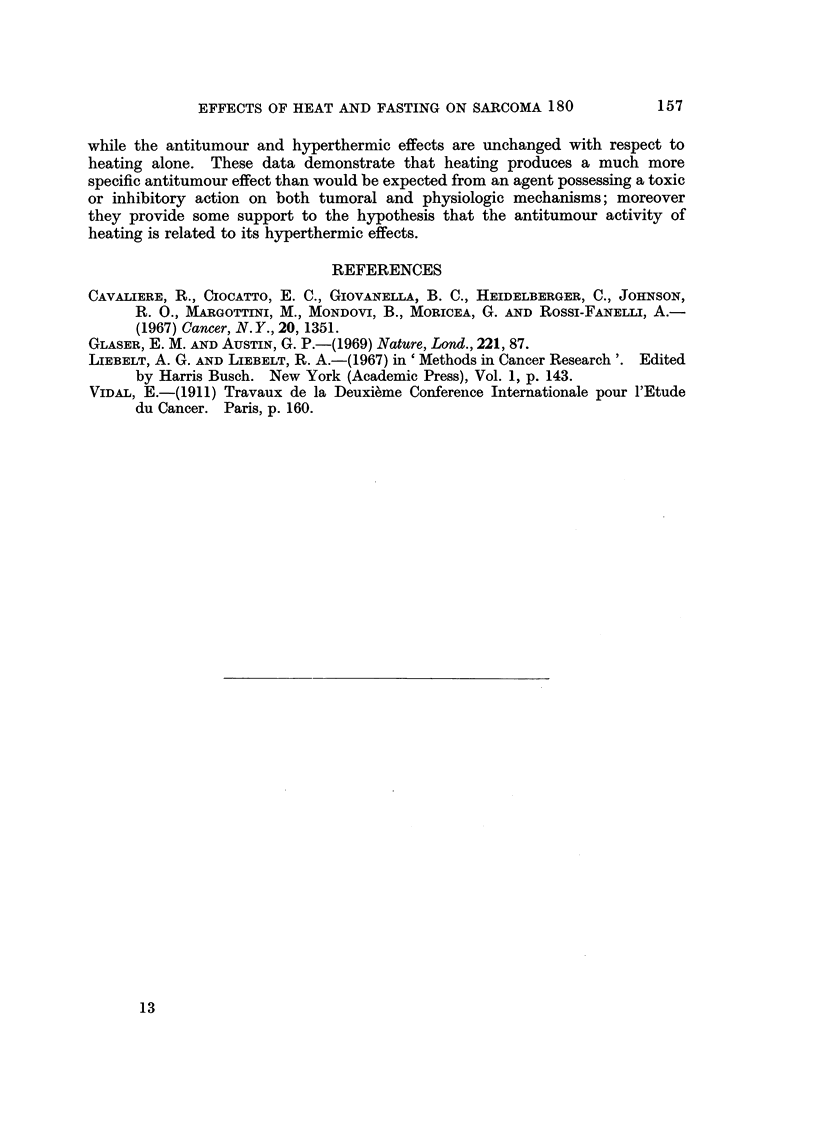# Comparative Effects of Heating and Fasting in Mice, with Particular Reference to Development of Sarcoma 180

**DOI:** 10.1038/bjc.1971.21

**Published:** 1971-03

**Authors:** V. Cioli, B. Silvestrini

## Abstract

Effects of heating and fasting, both alone and associated, have been studied in normal and Sarcoma 180 bearing mice. Heating reduced body weight and tumour mass and increased body temperature. Fasting reduced body weight, while tumour mass and body temperature were slightly affected. By associating heating and fasting a more marked decrease of body weight was produced than by each of the two factors involved, while effects on body temperature and on tumour mass were unchanged with respect to heating alone. 6-mercaptopurine was similar to heating in reducing body weight and tumour mass.


### COMPARATIVE EFFECTS OF HEATING AND FASTING IN MICE, WITH PARTICULAR REFERENCE TO DEVELOPMENT OF SARCOMA 180

V. CIOLI AND B. SILVESTRINI

*From the Department of Pharmacology, Research Laboratories, A.C.R. Angelini F., Rome, Italy*

Received for publication November 25, 1970

**SUMMARY.—Effects of heating and fasting, both alone and associated, have been studied in normal and Sarcoma 180 bearing mice. Heating reduced body weight and tumour mass and increased body temperature. Fasting reduced body weight, while tumour mass and body temperature were slightly affected. By associating heating and fasting a more marked decrease of body weight was produced than by each of the two factors involved, while effects on body temperature and on tumour mass were unchanged with respect to heating alone. 6-mercaptopurine was similar to heating in reducing body weight and tumour mass.**

SEVERAL clinical and laboratory reports, which have been reviewed by Cavaliere *et al.* (1967), indicate that infections with concomitant high fever may lead to disappearance of tumours in cancer patients; they also provide some evidence relating these findings to the liability *in vitro* of cancer cells to high temperatures.

The study of tumour growth in animals maintained at high temperature led to contrasting results. Vidal (1911) observed that mice bearing transplanted tumours survived longer when they were maintained at elevated than at room temperature, while Glaser and Austin (1969) reported no statistical difference between tumour weights of mice maintained at 33·5 or 22·5° C. Our experiments were aimed at investigating in greater detail the influence of high temperature on mice bearing Sarcoma 180 and comparing the effects of heating with the effects of fasting and 6-mercaptopurine.

### MATERIAL AND METHODS

Female CF1 mice, 50 days old, were used and kept isolated throughout all the experiments. Pieces of solid Sarcoma 180, weighing about 35 mg., were subcutaneously implanted in the interscapular region. The two largest dimensions of the tumours were determined and the products of these two numbers were used as an index of tumour mass, according to Liebelt and Liebelt (1967). Index of tumour mass has been found to be roughly correlated to tumour weight expressed in g. Animals were usually kept at a temperature of 22° C. Experiments were started 7 days following implantation of Sarcoma 180.

Mice showing no tumour growth were discarded and homogeneous groups of mice were prepared according to the tumour mass. Body temperature was measured by an Ellab apparatus, mod. TE 3, inserting the thermocouples 3 cm. deep into the rectum.

The influence of the following factors, on both normal and Sarcoma 180 bearing mice, was studied:

(a) *Heating.* Heating tests were performed by changing the room temperature according to the following scheme: 35° C. on the 1st and 2nd days; 36° C. on the 3rd and 4th days; 37° C. on the 5th–14th days; 32° C. on the 15th day; 28° C. on the 16th day; 26° C. on the 18th day; 22° C. on the following days.

(b) *Fasting.* Fasting tests were performed by depriving animals of food, but not of water, every second day for 14 days.

(c) *Heating and fasting associated.* Mice were submitted at the same time to heating and fasting, according to the above described procedures.

(d) 6-*mercaptopurine.* The drug was subcutaneously injected daily, for 7 days, away from the tumour implantation site. The compound was suspended in 0·5 per cent methylcellulose and its concentrations were adjusted in order to give a volume of 10 ml./kg. Control animals received the same volume of a 0·5 per cent methylcellulose suspension.

The significance of results was assessed according to the Student's t test.

### RESULTS

Table I illustrates the effects of heating on Sarcoma 180 bearing and normal mice.

Heating produced a significant inhibition of both body weight and tumour mass in Sarcoma 180 bearing mice.

In normal mice heating affected body weight to a lesser extent than in tumour-bearing animals.

TABLE I.—*Effects of Heating on Sarcoma 180-Bearers and Normal Mice*

| | | Time in weeks | | | | |
|---|---|---|---|---|---|---|
| | | 0 | 1 | 2 | 3 | 4 |
| A | No. of mice | 55 | 54 | 54 | 52 | 52 |
| Bearers of Sarcoma 180 | Body weight | 25·29 ±0·39 | 26·35 ±0·37 | 28·35 ±0·47 | 31·64 ±0·63 | 36·04 ±0·91 |
| | Index of tumour mass | 0·71 ±0·02 | 1·25 ±0·07 | 2·43 ±0·20 | 4·74 ±0·43 | 7·73 ±0·70 |
| B | No. of mice | 55 | 53 | 43 | 40 | 40 |
| Bearers of Sarcoma 180 submitted to elevated temperatures | Body weight | 25·71 ±0·32 | 23·32* ±0·22 | 22·95* ±0·34 | 27·51* ±0·37 | 31·73* ±0·58 |
| | Index of tumour mass | 0·71 ±0·02 | 0·85* ±0·05 | 1·25* ±0·15 | 2·08* ±0·28 | 4·36* ±0·58 |
| C | No. of mice | 17 | 17 | 17 | 17 | 17 |
| Normal mice | Body weight | 25·70 ±0·73 | 25·32 ±0·66 | 26·11 ±0·59 | 26·47 ±0·72 | 27·73 ±0·67 |
| D | No. of mice | 17 | 13 | 9 | 9 | 9 |
| Normal mice submitted to elevated temperatures | Body weight | 25·70 ±0·64 | 23·62 ±0·59 | 22·23* ±0·70 | 25·39 ±0·63 | 26·34 ±0·70 |

Means ± SE are given.
Group B was compared to group A, and group D to group C.
* $P < 0{\cdot}001$.

TABLE II.—*Effects of Fasting on Sarcoma 180-Bearers and Normal Mice*

| | | Time in weeks | | | | |
|---|---|---|---|---|---|---|
| | | 0 | 1 | 2 | 3 | 4 |
| A Bearers of Sarcoma 180 | No. of mice | 46 | 46 | 46 | 44 | 44 |
| | Body weight | 26·43 ±0·36 | 27·30 ±0·35 | 29·53 ±0·50 | 33·01 ±0·64 | 37·02 ±0·94 |
| | Index of tumour mass | 0·72 ±0·02 | 1·32 ±0·09 | 2·60 ±0·24 | 4·81 ±0·51 | 7·70 ±0·81 |
| B Bearers of Sarcoma 180 submitted to fasting | No. of mice | 46 | 46 | 46 | 46 | 46 |
| | Body weight | 26·04 ±0·33 | 22·66* ±0·34 | 27·01* ±0·45 | 31·05* ±0·51 | 35·65 ±0·83 |
| | Index of tumour mass | 0·72 ±0·02 | 1·01† ±0·08 | 2·13 ±0·23 | 4·58 ±0·50 | 7·98 ±0·85 |
| C Normal mice | No. of mice | 17 | 17 | 17 | 17 | 17 |
| | Body weight | 25·70 ±0·73 | 25·32 ±0·66 | 26·11 ±0·59 | 26·47 ±0·72 | 27·73 ±0·67 |
| D Normal mice submitted to fasting | No. of mice | 17 | 17 | 17 | 17 | 17 |
| | Body weight | 25·70 ±0·76 | 20·03* ±0·51 | 24·35‡ ±0·60 | 25·29 ±0·64 | 26·94 ±0·61 |

Means ± SE are given.
Group B was compared to group A, and group D to group C.
*$P < 0·001$.
†$P < 0·01$.
‡$P < 0·05$.

TABLE III.—*Combined Effects of Heating and Fasting on Sarcoma 180-Bearers and Normal Mice*

| | | Time in weeks | | | | |
|---|---|---|---|---|---|---|
| | | 0 | 1 | 2 | 3 | 4 |
| A Bearers of Sarcoma 180 | No. of mice | 28 | 28 | 28 | 26 | 26 |
| | Body weight | 26·75 ±0·45 | 27·32 ±0·46 | 28·87 ±0·71 | 32·73 ±0·95 | 36·94 ±1·34 |
| | Index of tumour mass | 0·76 ±0·03 | 1·36 ±0·10 | 2·50 ±0·28 | 5·02 ±0·68 | 7·88 ±1·05 |
| B Bearers of Sarcoma 180 submitted to heating and fasting | No. of mice | 28 | 27 | 24 | 24 | 24 |
| | Body weight | 26·55 ±0·40 | 21·18* ±0·34 | 22·97* ±0·48 | 28·10* ±0·52 | 32·50† ±0·84 |
| | Index of tumour mass | 0·76 ±0·02 | 0·86* ±0·07 | 1·21* ±0·18 | 2·33† ±0·42 | 4·75‡ ±0·77 |
| C Normal mice | No. of mice | 17 | 17 | 17 | 17 | 17 |
| | Body weight | 25·70 ±0·73 | 25·32 ±0·66 | 26·11 ±0·59 | 26·47 ±0·72 | 27·73 ±0·67 |
| D Normal mice submitted to heating and fasting | No. of mice | 17 | 15 | 12 | 12 | 12 |
| | Body weight | 25·70 ±0·67 | 19·51* ±0·52 | 20·47* ±0·52 | 25·26 ±0·78 | 27·43 ±0·67 |

Means ± SE are given.
Group B was compared to group A, and group D to group C.
*$P < 0·001$.
†$P < 0·01$.
‡$P < 0·05$.

**Fig. 1.—Effects of heating and fasting, both alone and combined, on tumour mass of Sarcoma 180-bearers.**

**Fig. 2.—Effects of heating and fasting, both alone and combined, on body weight of normal mice. Each group consisted of 17 mice.**

Table II illustrates effects of fasting on Sarcoma 180 bearing and normal mice.

Tumour-bearers presented, under the influence of fasting, a more evident decrease of body weight than of tumour mass. Fasting produced a significant decrease of body weight also in normal mice.

Table III summarized results obtained by studying the combined effects of heating and fasting in tumour-bearing and normal mice.

Heating and fasting reduced both body weight and tumour mass.

Body weight was decreased by the combined effects of heating and fasting also in normal mice.

FIG. 3.—Effects of heating and fasting, both alone and combined, on body weight of Sarcoma 180-bearers. Each group consisted of 8 mice.

Fig. 1 summarizes results of the above experiments in connection with effects on tumour mass of heating and fasting, both alone and associated.

Plotting of results clearly shows that the antitumour activity of heating is not enhanced by simultaneous fasting of animals.

To study in deeper details the influence of heating and fasting on body weight, a number of experiments were duplicated by conducting daily determinations instead of weekly ones. Fig. 2 compares the curves of body weight in normal mice submitted to heating and fasting, both alône and associated.

FIG. 4.—Effects of heating and fasting, both alone and combined, on body temperature of normal mice. Each group consisted of 17 mice.

In agreement with data previously presented, both groups submitted to heating or fasting present a decrease of body weight. By combining heating and fasting a more marked effect is observed than with either of the two factors alone. With respect to weekly determinations, the daily ones provide additional information only in the case of fasting, whose effects present marked daily variations.

Fig. 3 illustrates the curves of body weight daily determined in tumour-bearing animals submitted to heating and fasting, both alone and combined.

Both groups submitted to heating or fasting presented a decrease of body weight which, however, quite contrary to that observed in normal mice, is particularly evident under the influence of heating. By combining heating and fasting a more marked decrease of body weight is observed than under the influence of either factor alone. In this experiment also, the performing of daily determinations appears to furnish relevant information only in the case of fasting.

Fig. 4 illustrates the curves of body temperatures recorded in normal mice submitted to heating or fasting, both alone and associated.

Heating produced a hyperthermic response, which was substantially similar when fasting was associated with heating. Fasting alone produced, particularly at the beginning of the experiment, a hypothermic reaction every second day corresponding to withdrawal of food.

FIG. 5.—Effects of heating and fasting, both alone and combined, on body temperature of Sarcoma 180-bearers. Each group consisted of 17 mice.

Fig. 5 compares the curves of body temperatures recorded in tumour-bearers submitted to heating or fasting, both alone and associated.

Results obtained are substantially similar to those recorded in normal mice.

Table IV illustrates effects of 6-mercaptopurine on Sarcoma 180-bearing mice. The treatment was given daily for 7 days. The results obtained show that at both the doses of 5 and 20 mg./kg. s.c., the drug reduced body weight and tumour mass. With the higher dose more than half of the animals died during the experiment.

TABLE IV.—*Effects of 6-Mercaptopurine on Sarcoma 180-Bearers (The drug was given daily for 7 days)*

| | | Time in weeks | | | | |
|---|---|---|---|---|---|---|
| | | 0 | 1 | 2 | 3 | 4 |
| Controls | No. of mice | 19 | 19 | 19 | 19 | 18 |
| | Body weight | 24·73 ±0·25 | 26·61 ±0·64 | 29·76 ±0·97 | 33·76 ±1·21 | 38·86 ±1·85 |
| | Index of tumour mass | 0·72 ±0·02 | 1·38 ±0·12 | 3·61 ±0·41 | 6·73 ±0·70 | 10·14 ±1·07 |
| 6-mercaptopurine 5 mg./kg. s.c. | No. of mice | 19 | 19 | 19 | 19 | 19 |
| | Body weight | 24·34 ±0·42 | 24·74 ±0·54 | 27·26§ ±0·58 | 29·34† ±0·71 | 32·76† ±1·26 |
| | Index of tumour mass | 0·72 ±0·02 | 0·95‡ ±0·13 | 1·61* ±0·29 | 3·02* ±0·62 | 5·20† ±1·03 |
| 6-mercaptopurine 20 mg./kg. s.c. | No. of mice | 19 | 18 | 10 | 9 | 9 |
| | Body weight | 25·13 ±0·54 | 22·75* ±0·59 | 23·85* ±1·04 | 26·55* ±0·91 | 28·88* ±0·87 |
| | Index of tumour mass | 0·72 ±0·02 | 0·59* ±0·06 | 0·62* ±0·14 | 1·10* ±0·28 | 2·04* ±0·63 |

Means ± SE are given.

* $P < 0{\cdot}001$.

† $P < 0{\cdot}01$.

‡ $P < 0{\cdot}02$.

§ $P < 0{\cdot}05$.

### DISCUSSION

The main results of these experiments have been summarized in Table V.

TABLE V.—*Summary of Main Results Obtained in Normal and Tumour-Bearing Mice*

| | Normal mice | | Tumour-bearing mice | | |
|---|---|---|---|---|---|
| | Body weight decrease | Body temperature increase | Tumour mass inhibition | Body weight decrease | Body temperature increase |
| Heating | + | ++ | ++ | ++ | ++ |
| Fasting | + | − | ± | + | − |
| Heating and fasting | ++ | ++ | ++ | +++ | ++ |

Heating of normal mice reduced the body weight and produced an hyperthermic reaction. Tumour-bearers presented, under the influence of heating, a reduction of tumour mass increase, a hyperthermic reaction and a more marked decrease of body weight than normal mice. Fasting reduced the body weight to the same extent in normal and tumour-bearing animals. Moreover fasting did not produce any hyperthermic reaction and its antitumour activity was slight and of short duration. By associating heating and fasting, a summation of effects has been observed at the level of body weight decrease, while the hyperthermic and anti-tumour activity of heating was unchanged.

On the whole, these results demonstrate that heating possesses an antitumour activity and provide some indication regarding its significance. In this connection, the following data must be taken into account. Fasting reduces body weight, while its effects on tumour mass are insignificant; by associating heating and fasting a more marked inhibition of body weight is observed than the two factors isolated, while the antitumour and hyperthermic effects are unchanged with respect to heating alone. These data demonstrate that heating produces a much more specific antitumour effect than would be expected from an agent possessing a toxic or inhibitory action on both tumoral and physiologic mechanisms; moreover they provide some support to the hypothesis that the antitumour activity of heating is related to its hyperthermic effects.

### REFERENCES

Cavaliere, R., Ciocatto, E. C., Giovanella, B. C., Heidelberger, C., Johnson, R. O., Margottini, M., Mondovi, B., Moricea, G. and Rossi-Fanelli, A.—(1967) *Cancer, N.Y.*, **20**, 1351.

Glaser, E. M. and Austin, G. P.—(1969) *Nature, Lond.*, **221**, 87.

Liebelt, A. G. and Liebelt, R. A.—(1967) in ' Methods in Cancer Research '. Edited by Harris Busch. New York (Academic Press), Vol. 1, p. 143.

Vidal, E.—(1911) Travaux de la Deuxième Conference Internationale pour l'Etude du Cancer. Paris, p. 160.